# Dielectric Barrier Discharge Plasma Coupled with Cobalt Oxyhydroxide for Methylene Blue Degradation

**DOI:** 10.3390/toxics11090763

**Published:** 2023-09-08

**Authors:** Xiaomei Yao, Yingbo Fang, Xiaochen Cui, Xian Cheng, Zixia Cheng

**Affiliations:** 1 School of Electrical and Information Engineering, Zhengzhou University, Zhengzhou 450001, China; xm_yao@zzu.edu.cn (X.Y.); 16696658179@163.com (Y.F.); 13031652377@163.com (X.C.);; 2 Henan Engineering Research Center of Power Transmission & Distribution Equipment and Electrical Insulation, Zhengzhou University, Zhengzhou 450001, China

**Keywords:** double dielectric barrier discharge (DDBD), MB degradation, CoOOH, degradation performance

## Abstract

In this study, the coupled use of a double dielectric barrier discharge (DDBD) and CoOOH catalyst was investigated for the degradation of methylene blue (MB). The results indicated that the addition of CoOOH significantly promoted MB degradation performance compared to the DDBD system alone. In addition, both the removal rate and energy efficiency increased with an increase in CoOOH dosage and discharge voltage. After 30 min of discharge treatment in the coupled system (with CoOOH of 150 mg), the removal rate reached 97.10% when the discharge voltage was 12 kV, which was 1.92 times that in the single DDBD system. And when the discharge time was 10 min, the energy efficiency could reach 0.10 g (k·Wh)^−1^, which was 3.19 times better than the one in the single DDBD system. Furthermore, the addition of CoOOH could also significantly enhance the TOC and COD removal rates of MB. In the DDBD-coupled-with-CoOOH system, TOC and COD were 1.97 times and 1.99 times those of the single DDBD system after 20 min of discharge treatment with a discharge voltage of 12 kV and 100 mg of CoOOH. The main active substances detected in the coupled system indicated the conversion of the active species H_2_O_2_ and O_3_ into a more oxidizing ·OH was enhanced through the addition of a CoOOH catalyst, resulting in the more effective decomposition of MB and intermediate molecules.

## 1. Introduction

In the textile industry, various organic synthetic dyes are commonly used as coloring materials. Because of their variety and complex structure, synthetic dyes are a kind of complex environmental pollutant, which poses a huge ecological risk to the environment [1]. Dyes enter water systems and cause the death of aquatic species and microorganisms, posing a serious threat to ecosystems and human health [2]. Synthetic dyes are relatively stable compounds that are difficult to degrade in wastewater treatment based on physical, chemical, or/and biological treatments. Therefore, developing an effective wastewater treatment process to remove dyes has been the focus.

Advanced oxidation processes (AOPs) have gained attention in recent years in the treatment of dye contaminants, such as photochemical, catalytic oxidation, ozonation, and Fenton or photo-Fenton systems. However, some problems, such as the addition of additional chemical reagents and unsatisfactory energy efficiency, still exist in these AOP systems [3]. As a comprehensive AOP technology, plasma has become a feasible technology for decomposing these pollutants effectively. In the plasma method, energy is injected into an aqueous solution through a high-voltage discharge plasma channel between two electrodes, and it produces various active species such as ·OH, ·O, O_3_, and H_2_O_2_ in the aqueous solution with high oxidation–redox potential for the decomposition of the target molecule [4].

Among these active substances, ·OH exhibits a high oxidation potential and is recognized as the most effective active substance for degrading pollutant molecules. Therefore, how to make full use of various active substances generated by plasma, especially those with a relatively low oxidation potential (such as O_3_ and H_2_O_2_), and increase the ·OH yield has become the focus of research. The addition of catalysts, such as adding an iron agent into plasma system for a Fenton effect, can realize the conversion of H_2_O_2_ generated in situ to ·OH and then maximize the utilization of various generated active substances in the plasma. Therefore, compared to a separate discharge plasma system, the plasma–catalytic system has the advantages of both good degradation performance and high energy utilization in catalytic reactions, making it an effective technology. Commonly used heterogeneous catalysts, which are conducive to recovery, mainly include transition metal oxides, such as TiO_2_ [5], ZnO [6], WO_3_, etc. In recent years, studies have shown that hydroxyl metal oxide is a promising type of catalyst with good catalytic performance, abundant properties, and good stability [7]. Hydroxyl metal oxides exhibit high specific surface area, which can provide sufficient active sites for catalyst reactions, and are also beneficial for complex multi-electron and multi-phase reaction process [7,8]. Zhao et al. compared the catalytic effects of FeOOH and various iron oxides in Fenton-like reactions, and found that FeOOH can more effectively catalyze the degradation of organic pollutants in water, and the conversion efficiency of H_2_O_2_ to ·OH is also the highest [8]. Additionally, cobalt is an ideal metal catalyst owing to its excellent catalytic performance and low ion leakage, arousing a wide range of research interests [9,10]. Xu et al. has successfully employed cobalt hydroxide in the decomposition of ozone and p-chloronitrobenzene (pCNB) in water [11]. Ren et al. introduced a CoOOH catalyst into a plasma system for the degradation of trans-ferulic acid wastewater, and the results showed good degradation performance. It has been concluded that the H_2_O_2_ and O_3_ generated in the discharge plasma are first decomposed into peroxide-like oxides at the active site of the catalyst and then converted into ·OH with higher oxidation ability to achieve the efficient degradation of the target [12].

Inspired by this, we introduced a CoOOH catalyst into a gas–liquid hybrid double dielectric barrier discharge (DDBD) for the degradation of dye in wastewater. DDBD devices, which usually employ an additional dielectric barrier to isolate the metal electrode from the solution to avoid corrosion, have been widely used in water treatment [13,14,15,16]. The specific DDBD system can be reflected in our previous research work [17]. In this paper, we chose methylene blue (MB) as the target, which is a model azo dye with a stable molecular structure, and it is difficult to degrade using traditional methods [18,19,20]. The physical and chemical properties of CoOOH in the plasma–catalytic system were characterized via XRD, EDX, XPS, and SEM. The MB degradation performance including MB degradation efficiency, energy efficiency, COD, and TOC was evaluated. Additionally, the effect of different operating parameters on MB degradation were investigated. Moreover, the catalytic mechanisms of CoOOH in this discharge plasma system were explored.

## 2. Materials and Methods

### 2.1. Materials

Cobaltous nitrate (Co(NO_3_)_2_·6H_2_O) and methylene blue (99.99%) were purchased from Shanghai Aladdin Biochemical Technology Co., Ltd., Shanghai, China, and Tianjin Zhiyuan Chemical Reagent Co., Ltd., Tianjin, China, respectively. Sodium indigo disulphonate and potassium permanganate were purchased from Sinopharm Chemical Reagent Co., Ltd., Shanghai, China. Salicylic acid, 2,3-dihydroxybenzoic acid, and 2,5-dihydroxybenzoic acid were purchased from Aladdin. Besides these, other reagents used in this study were analytical grade, and no further purification was required. Deionized water was used in all experiments.

### 2.2. Catalyst Preparation

The CoOOH catalyst was prepared according to the method in previous work [17]. An amount of 4.3 g of Co(NO_3_)_2_·6H_2_O was completely dissolved in 250 mL deionized water, and then an H_2_O_2_ solution with a concentration of 30% was added. The mixed liquid was stirred at a constant temperature under 55 °C with a magnetic stirrer. During the stirring process, 0.5 mol L^−1^ NaOH solution was added to adjust the pH value of the mixed solution to 9~10, and then, it was stirred at a constant temperature for more than 1 h for a uniform solution. The solution containing the CoOOH catalyst was then repeatedly washed and separated at least three times using an accelerated centrifuge. Finally, it was dried at 70 °C, and the dried powder was crushed and sifted (<35 μm). Thus, CoOOH catalyst powder could be obtained.

The crystal phases of the samples were evaluated via X-ray powder diffraction (XRD, Brucker D8 Advance, Broker Scientific Instruments, Billerica, MA, USA) in the range of 10–90° (2θ), using Cu Kα radiation. The morphologies of the obtained samples were characterized using a scanning electron microscope (SEM, Hitachi, SU8020, Tokyo, Japan). X-ray photoelectron spectroscopy (XPS) was performed on a Thermo escalab 250Xi (Thermo escalab, Carlsbad, CA, USA).

### 2.3. Experimental Setup

**Figure 1:**
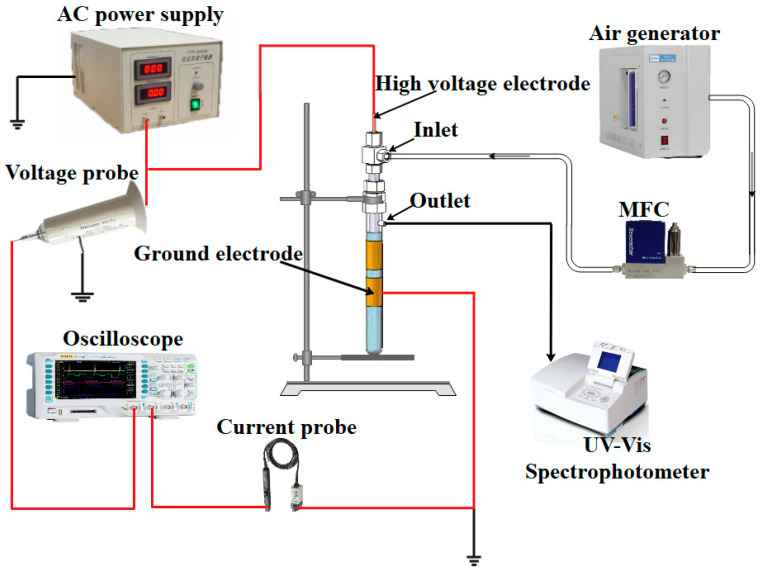
Schematic diagram of experimental setup.

The gas–liquid hybrid double dielectric barrier discharge (DDBD) reactor used in this study was the same as in our previous work [17]. A copper rod (8 mm in diameter and 220 mm in length) was used as the high-voltage electrode. Two quartz tubes were used as the insulating medium, with a gap of 1 mm. The inner one, with an inner diameter of 9 mm and an outer diameter of 12 mm, was covered outside the copper rod electrode, and the gap between them was 1.5 mm. The inner and outer diameter of the outer quartz tube were 18 mm and 20 mm, respectively. Four identical copper strips connected in series with a total length of 80 mm, which were attached tightly to the outside surface of the outer quartz tube, were used as the grounding electrode. The DDBD plasma reactor was supplied by a high-frequency AC power supply (CTP-2000K, Nanjing Suman Electronics Co., Ltd., Nanjing, China). The voltage frequency was 10.13 kHz. A high-voltage probe (Tektronix P6015A, Cincinnati, OH, USA) and a voltage probe (Rigol PVP2150, Suzhou, China) were employed to record the voltage and current delivered to the reactor. The schematic diagram of the experimental setup is shown in Figure 1.

A mass flow meter (MFC, D07, Beijing Seven star Flow Co., Ltd., Beijing, China) was employed to control the compressed air from the air generator flowing into the DDBD reactor. Compressed air was injected through the air inlet into the zone between the copper rod and the inner quartz tube, forming a gas-phase discharge under a strong electric field. Then, the discharged gas was injected into the MB solution between two dielectric tubes with an aerator for the following chemical reaction process.

### 2.4. Methods and Analysis

A UV-vis spectrophotometer (TU-1810, Puxi, Beijing, China) was used to detect the concentration of the MB solution. The calculation methods of removal efficiency, energy efficiency, and the first-order reaction kinetics model were similar to our previous work [17]. The dissolved O_3_ centration (mg L^−1^) in the liquid phase was measured and calculated using indigo disulfonate spectrophotometry (IDS) [21], the H_2_O_2_ concentration (mg L^−1^) in the MB solution was determined using titanium sulfate spectrophotometry, and the ·OH concentration (mol L^−1^) in the MB solution was determined via the salicylic acid probe method. TOC and COD were determined using the Analytik Jena Mutiny N/C 2100 (Jena, Germany) and HQ40D-Multi (hach, Loveland, CO, USA), respectively. In addition, unless otherwise specified, the volume of the MB solution in the experiment was 30 mL, the air flow rate was 50 mL min^−1^, and the concentration of the MB solution was 50 mg L^−1^.

## 3. Results

### 3.1. Characterization of CoOOH

**Figure 2:**
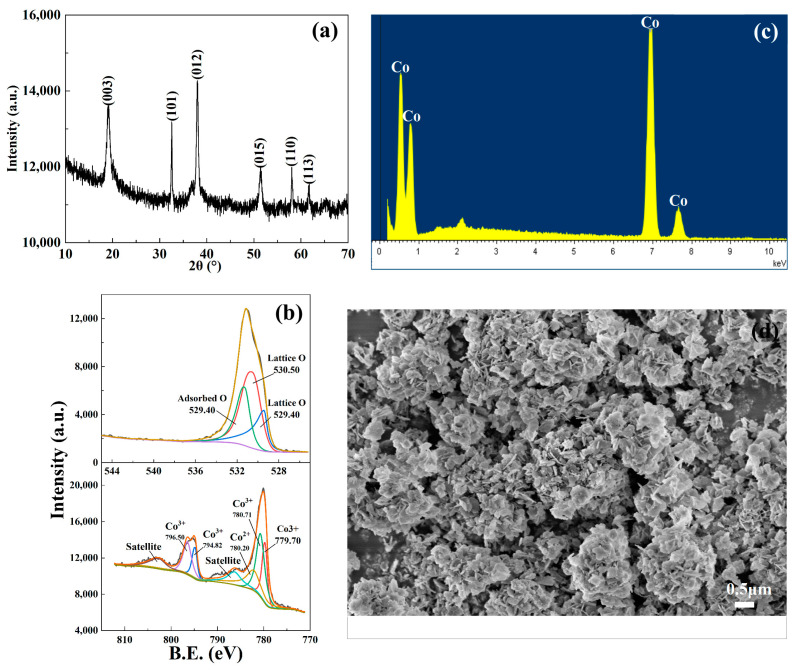
(**a**) XRD, (**b**) XPS, (**c**) SED, and (**d**) SEM of the CoOOH catalyst.

XRD is a crucial method for investigating the crystal structure of substances. Through analyzing the XRD diffraction pattern using Jade 9 software, the valuable information about the crystal structure of the catalyst used was obtained. In Figure 2a, it can be observed that CoOOH exhibited six diffraction peaks at specific angles: 20.2°, 36.9°, 38.8°, 50.7°, 65.3°, and 69.1°. Through a comparison of the diffraction data with the PDF4-2009 card database, it was determined that these peaks correspond to the crystal phases 003, 101, 012, 015, 110, and 113 of CoOOH, respectively.

XPS was employed to analyze the valence distribution of each element in the catalyst. In Figure 2b, the peaks at 529.40 eV and 530.5 eV represent oxygen atoms within the lattice of the substance [22,23], and the peak at 531.28 eV indicates oxygen atoms from ·OH adsorbed on the surface of the measured substance [24,25,26,27]. It is calculated that the lattice oxygen atoms account for 33.28% of the total number of atoms, and the oxygen atoms from the adsorbed ·OH account for 13.39% of the total. This ratio is consistent with the expected proportion of lattice oxygen atoms and adsorbed oxygen atoms in CoOOH. Additionally, the XPS energy spectrum of Co2p was analyzed. The peaks of Co^3+^ appeared at 779.70 eV, 780.71 eV, 794.82 eV, and 796.50 eV, and the peak of Co^2+^ appeared at 782.20 eV [27,28]. Additionally, EDS was utilized to detect the catalyst, enabling the quantitative analysis of each element. As depicted in Figure 2c, distinct characteristic peaks of Co and O are detected, with a molecular ratio of approximately 36.52% for Co and 63.48% for O, which closely matches the theoretical ratio for CoOOH. Consequently, there is strong evidence supporting the identification of the catalyst as CoOOH.

The surface morphology of CoOOH was examined via SEM, and the particle size was calculated. Figure 2d presents an enlarged SEM image of CoOOH, revealing its relatively rough surface with a typical layered structure. The particle size distribution is fairly uniform, with the majority of particles concentrated within 4.5 μm, which could ensure that CoOOH has a considerable contact area with the active substances.

### 3.2. MB Degradation Performance via DDBD with CoOOH Catalyst

#### 3.2.1. Effect of CoOOH Dosage on MB Degradation

**Figure 3:**
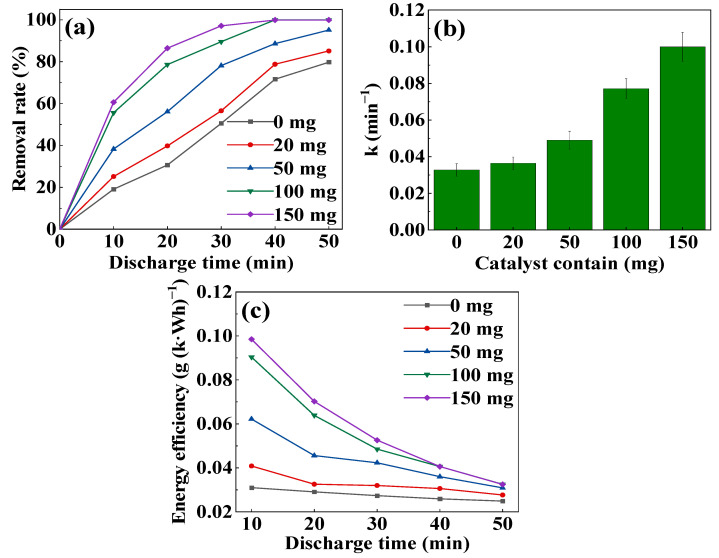
MB degradation under different dosages of CoOOH catalyst: (**a**) removal rate, (**b**) first-order reaction rate constant, (**c**) energy efficiency (discharge voltage of 12 kV).

In Figure 3a,b, it is evident that with the same duration of discharge treatment, both the removal rate and first-order reaction rate constant increase as the dosage of CoOOH catalyst increases. After 30 min of discharge treatment, without CoOOH, the removal rate of MB was only 50.52%. However, when the dosages of CoOOH were 100 mg and 150 mg, the removal rates increased to 89.54% and 97.10%, respectively, which were 1.77 times and 1.92 times that without CoOOH, respectively. Meanwhile, the first-order reaction rate constants rose from 0.03 min^−1^ to 0.08 min^−1^ and 0.10 min^−1^, which were 2.33 times and 3.03 times that in the single DDBD system. Furthermore, after 40 min of discharge, the removal rate of MB reached 99.99% (12 kV, 100 mg of CoOOH). These results indicate that the inclusion of CoOOH significantly promotes the removal rate and reaction rate of MB degradation. Therefore, the DDBD-coupled-with-CoOOH system exhibits a distinct advantage over DDBD alone for the degradation of MB.

From Figure 3c, it can be observed that the energy efficiency gradually increases with the increase in CoOOH dosage after the same discharge treatment time, while decreases as the discharge time prolongs. Without CoOOH, the energy efficiency was only 0.03 g (k·Wh)^−1^ after 10 min, and it decreased slowly with time. However, when 100 mg and 150 mg of CoOOH were added, respectively, the energy efficiency reached 0.09 g (k·Wh)^−1^ and 0.1 g (k·Wh)^−1^, respectively, which were 2.90 times and 3.19 times of that without CoOOH. Additionally, the energy efficiency decreased significantly as the discharge time extended.

Some researches indicated the CoOOH facilitated the formation of peroxide-like compounds on its surface by the active substances generated during the discharge process [29,30]. Additionally, the addition of CoOOH in this DDBD plasma system can significantly improve the MB degradation performance, which may attribute to the abundant ·OH on the catalyst surface and the conversion process is discussed in detail in Section 3.3. Although more active substances were generated as the discharge time extends, most MB molecules were degraded into smaller molecules in the early stage, reducing the average collision probability with active substances, which lead to a continuous decline of energy efficiency.

#### 3.2.2. Effect of Discharge Voltage on MB Degradation

**Figure 4:**
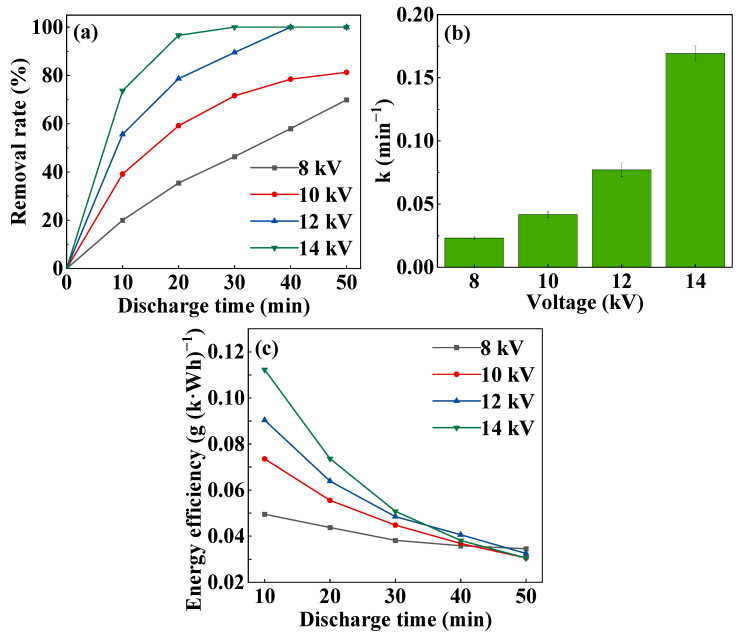
MB degradation under different discharge voltages: (**a**) removal rate, (**b**) first-order reaction rate constant, (**c**) energy efficiency (the dosage of CoOOH was 100 mg).

From Figure 4a,b, it is obvious that in the DDBD plasma coupled with CoOOH system, the MB removal rate and first-order reaction rate constant increase with the increase in discharge voltage. After 20 min of discharge treatment, when the discharge voltage increased from 8 kV to 14 kV, the MB removal rate rose from 35.43% to 96.61%. Meanwhile, the first-order reaction rate constant increased from 0.02 min^−1^ to 0.18 min^−1^. This was because as the discharge voltage increased, more energy was injected into the reactor, causing MB to degrade more in a limited time. In the DDBD system alone, after 40 min of discharge treatment and the discharge voltage was 12 kV, the MB removal rate and first-order reaction rate constant were 71.62% and 0.03 min^−1^, respectively. However, in the system comprising DDBD plasma coupled with CoOOH, the MB was almost completely degraded and the energy efficiency reached 0.09 min^−1^, which was 1.33 times higher than that of without CoOOH. In brief, the addition of the catalyst CoOOH significantly enhances the removal rate and first-order reaction rate constant.

Figure 4c shows the energy efficiency of MB degradation. It can be seen that the energy efficiency decreases with the extension in time at the same voltage. At 14 kV of discharge voltage, when the discharge time increased from 10 min to 30 min, the energy efficiency decreased from 0.11 g (k·Wh)^−1^ to 0.05 g (k·Wh)^−1^. This was due to the higher concentration of MB in the initial solution, the larger average contact area between MB molecules and active particles, which lead to the higher probability of high-energy particles colliding with MB molecules. However, with the increase in discharge time, more and more MB molecules were gradually oxidized into smaller molecules, resulting in a huge decrease in the number of MB molecules that could be oxidized per unit time in the subsequent time, so the energy efficiency decreased over time. For the same discharge time, the energy efficiency increased with the increase in discharge voltage. After discharge treatment for 10 min, it was 0.11 g (k·Wh)^−1^ at a discharge voltage of 14 kV, which was 2.20 times that of 8 kV. In the discharge process, in addition to the production of more active substances, the O_3_ and H_2_O_2_ that are generated would not only be decomposed into peroxide-like compounds on the surface of CoOOH [31], but also be converted into ·OH under the catalysis of CoOOH, resulting in an exceptionally high MB removal rate. The increase in discharge voltage greatly promoted the conversion of this process, so the energy efficiency increased with the increase in discharge voltage. After 30 min, due to the large reduction in MB molecules contained in the solution, even if a voltage of 14 kV produces more active substances, the number of MB molecules that could be degraded per unit time was greatly reduced, resulting in a decrease in energy efficiency, even less than the energy efficiency at a voltage of 8 kV.

#### 3.2.3. COD and TOC

**Figure 5:**
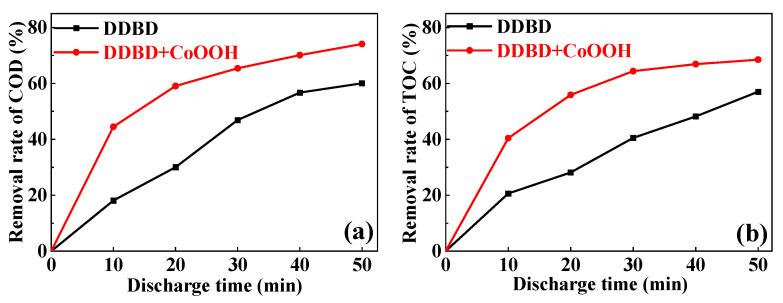
The removal rates of (**a**) COD and (**b**) TOC (discharge voltage of 12 kV, CoOOH dosage of 100 mg).

As shown in Figure 5, the chemical oxygen demand (COD) and total organic carbon (TOC) concentrations of MB solution under different discharge times were also measured. It can be seen that the removal rates of both COD and TOC gradually increase with the extension in discharge time, and in the DDBD-coupled-with-CoOOH system, they are significantly higher than in the single DDBD system. After 20 min of discharge treatment, the removal rates of COD and TOC in the DDBD system were 30.04% and 28.14%, respectively, while in the DDBD-coupled-with-CoOOH system, they reached 59.10% and 55.90%, respectively, which were 1.97 times and 1.99 times the former. Obviously, the addition of CoOOH significantly increases the MB mineralization rate. It may be due to the addition of CoOOH promoting the formation of ·OH, which exhibits stronger oxidation performance than H_2_O_2_ and O_3_, so that more MB and intermediates are degraded into small molecules per unit time, which will be analyzed in more detail in the following Section 3.3.

However, under the same conditions, the mineralization rate in the DDBD-coupled with-CoOOH-system is still lower than the removal rate. It can be seen from Figure 5 that after 40 min of discharge treatment, the removal rates of COD and TOC in the coupled system were 70.13% and 66.90%, respectively, while the removal rate is close to 100% in Figure 3a (100 mg CoOOH). It is due to the fact that during the plasma discharge process, the MB molecules are partially converted into small organic molecules or intermediates that are difficult to be mineralized, and only a part of the MB molecules are completely oxidized and decomposed into CO_2_ and H_2_O. MB is an azo dye whose azo bond is more likely to be broken than those of benzene and naphthalene rings [32]. Additionally, the azo bond is the chromogenic base of the azo dye, and most of the azo bond is broken during the degradation of MB, resulting in obvious the decolorization of the MB solution [33].

### 3.3. Mechanism of MB Degradation via DDBD Coupled with CoOOH

**Figure 6:**
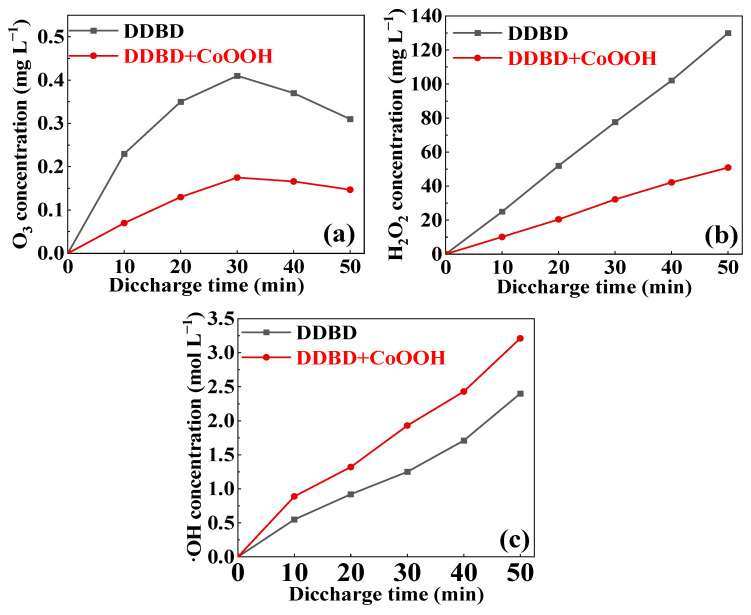
The dissolved concentration of (**a**) O_3_, (**b**) H_2_O_2,_ and (**c**) ·OH in MB solution (discharge voltage of 12 kV, CoOOH dosage of 100 mg).

In order to explore the mechanism of the synergistic effect between the DDBD and CoOOH, the content of active substances (H_2_O_2_, O_3_, ·OH) at different discharge times was detected. As shown in Figure 6a, in both systems, the O_3_ content initially increased and then decreased. It was due to the fact that in the discharge process, the air passed through the gas phase discharge region and produced active substances such as high-energy electrons and O_3_, and then these active substances entered the liquid phase with air to form e_(aq)_ and O_3(aq)_, as shown in Formulas (1)–(3). Therefore, at the beginning of the discharge, the O_3_ concentration in the solution gradually accumulated and increased with the extension in the discharge time. Until 30 min, the O_3_ concentration reached the maximum, 0.41 mg L^−1^ and 0.19 mg L^−1^ in both systems, respectively. However, after 30 min, the O_3_ content gradually decreased over time. In several studies, there was also an inflection point in the variation of dissolved O_3_ concentration with treatment time with a high-frequency power supply. It was due to the fact that the electrical discharge could heat up the temperature of the solution in these cases, which was adverse to the dissolution of O_3_ in water [5]. In contrast, it can be seen from Figure 6b,c that the H_2_O_2_ and ·OH contents continuously increased as the discharge time prolonged. This was mainly because the active substances dissolved in the solution underwent a series of oxidation reactions in the liquid phase to form H_2_O_2_ and ·OH, as shown in Formulas (4) and (5), and then these active substances (H_2_O_2_, ·OH) were gradually accumulated with the extension in time [34].

(1) $$O_{2(gas)}+e_{\left(gas\right)}\to O+O$$

(2) $$O+O_{2(gas)}+O_{2}\to O_{3(gas)}+O_{2}$$

(3) $$O+O_{2(aq)}+e_{aq}\to O_{3(aq)}$$

(4) $$O+H_{2}{O+e}_{aq}\to H_{2}O_{2}$$

(5) $$O+H_{2}O\to 2\cdot OH$$

Although the change trend of the active substance content in the two systems is similar, it can be observed from Figure 6 that the DDBD system had a higher content of O_3_ and H_2_O_2_, and a lower content of ·OH, compared to the DDBD-coupled-with-CoOOH system. This indicates that the addition of CoOOH changes the content of active substances in the solution. In other words, the addition of CoOOH promotes the formation of ·OH, which is a highly reactive specie with superior oxidation properties compared to O_3_ and H_2_O_2_ [29,35]. As a result, the MB removal rate, as well as COD and TOC removal rates, in the DDBD-coupled-with-CoOOH system are higher than those in the single DDBD system.

**Figure 7:**
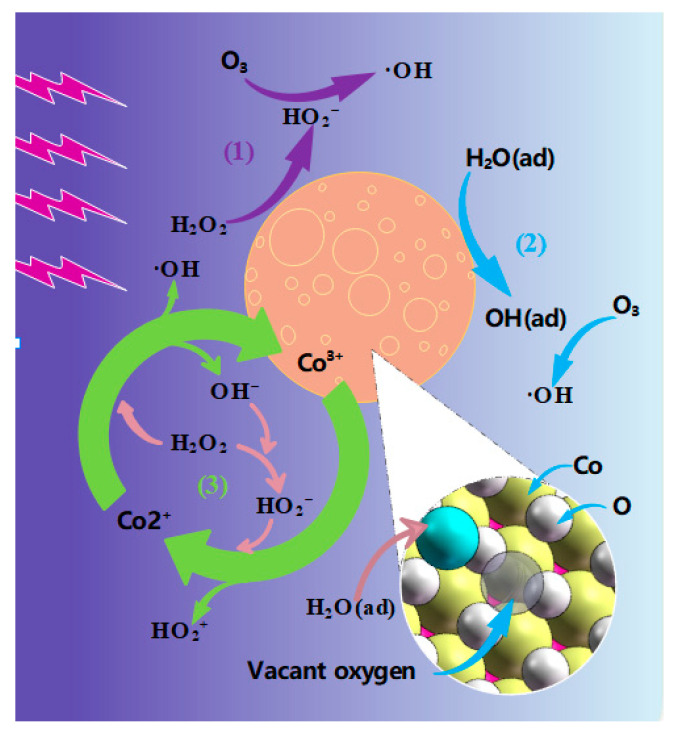
Mechanism of MB degradation via DDBD coupled with CoOOH.

Some studies have shown that there are abundant oxygen vacancies on the surface of CoOOH [36,37], and it is very easy for active substances such as H_2_O_2_ to form oxide-like compounds in the oxygen vacancies on the surface of CoOOH [38]; eventually, these oxide-like compounds react with O_3(aq)_ in the solution to form ·OH, such as in (6)~(11). The catalytic mechanism is shown in Figure 7 (route (1)). In addition, CoOOH has a large number of Lewis sites on its surface, which easily adsorb H_2_O molecules in contact with them and react with O_3(aq)_ in solution to form a ·OH group [39], as shown in Equations (12)–(14). These formed ·OH groups eventually dissociate from the CoOOH surface, leaving the Lewis site exposed again for the next reaction. This is the second way CoOOH promotes ·OH content, as shown in Figure 7 (route (2)). Furthermore, CoOOH exhibits efficient electron transfer through the Fenton reaction with oxides. First, Co^3+^ can oxidize with the generated oxide-like intermediates to produce ·OH and Co^2+^ [40,41,42]. Then, the H_2_O_2_ produced by the discharge react with Co^2+^ to produce Co^3+^, ·OH, and OH^−^, as shown in Formulas (15)–(17). The Co atom of CoOOH participates in this reaction to form ·OH, and a cycle is formed, which is shown in Figure 7 (route (3)).

(6) $$H_{2}O_{2}↔H^{+}+HO_{2}^{-}$$

(7) $$HO_{2}^{-}+O_{3(aq)}↔HO_{2}^{\cdot }+O_{3}^{\cdot -}$$

(8) $$HO_{2}^{\cdot }↔O_{2}^{\cdot -}+H^{+}$$

(9) $$O_{2}^{\cdot -}+O_{3}\to O_{2}+O_{3}^{\cdot -}$$

(10) $$O_{3}^{\cdot -}+H^{+}\to HO_{3}^{\cdot -}$$

(11) $$HO_{3}^{\cdot }\to \cdot OH+O_{2}$$

(12) $$O_{2}^{2-}(ad)+O_{3}+H_{2}O\to OH(ad)+HO_{3}^{+}+O_{2}$$

(13) $$OH(ad)+2O_{3}\to O_{2}^{2-}(ad)+HO_{3}^{-}+O_{2}$$

(14) $$HO_{3}^{-}\to \cdot OH+O_{2}$$

(15) $$H_{2}O_{2}+OH^{-}\to HO_{2}^{-}+H_{2}O$$

(16) $${Co}^{3+}+HO_{2}^{-}\to {Co}^{2+}+HO_{2}^{+}$$

(17) $${Co}^{2+}+H_{2}O_{2}\to {Co}^{3+}+\cdot OH+OH^{-}$$

## 4. Conclusions

In this study, CoOOH was added as a catalyst in a DDBD reaction system to achieve an effective removal rate and energy utilization efficiency when treating MB wastewater. The experimental results showed that, under a discharge voltage of 12 kV, an initial concentration of 50 mg L^−1^, an air flow rate of 50 mL min^−1^, and discharge treatment for 40 min, the removal rate of MB wastewater using the DDBD system with 100 mg CoOOH increased to 99.99%, which was 28.37% more than the DDBD system alone. Under the same conditions, the energy utilization efficiency increased to 0.04 g (k·Wh)^−1^, which was 1.57 times higher than that of the DDBD system alone, and the COD and TOC removal rates increased from 56.70% and 48.21% to 70.13% and 66.90%, respectively. This indicates that the addition of CoOOH could indeed achieve the purpose of improving the removal rate and energy efficiency of DDBDs and is expected to become a new efficient and environmentally friendly treatment method for MB wastewater.

## Data Availability

Data will be made available on request.
